# Secondary pallidonigral degeneration mimicking recurrent acute stroke in clinical presentation and magnetic resonance imaging: a case report

**DOI:** 10.1186/s12883-017-1000-5

**Published:** 2017-12-11

**Authors:** Ruei-Je Tsai, Li-Chun Hsieh, Sho-Jen Cheng, Cheng-Yu Chen

**Affiliations:** 1 Department of Medical Imaging, Taipei Medical University Hospital, 252 Wu Hsing Street, Taipei, 110 Taiwan; 2 Translational Imaging Research Center (TIRC), College of Medicine, Taipei Medical University, 252 Wu Hsing Street, Taipei, 110 Taiwan; 3 Department of Radiology, School of Medicine, College of Medicine, Taipei Medical University, 252 Wu Hsing Street, Taipei, 110 Taiwan

**Keywords:** Diffusion-weighted imaging, Pallidonigral degeneration, Recurrent stroke

## Abstract

**Background:**

Secondary pallidonigral transneuronal degeneration after a remote primary cerebral infarct can mimic recurrent stroke at clinical presentation. We describe a patient with secondary pallidonigral degeneration following a previous putaminal infarct, which was diagnosed through diffusion-weighted (DWI) and T2-weighted imaging (T2WI).

**Case presentation:**

A 64-year-old man complained of an acute relapse of right-lower-limb weakness following a cerebral infarction 2 months before presentation. Recurrent cerebral stroke was initially diagnosed in the emergency room. DWI of the brain revealed a subacute to chronic infarct in the left putamen and new acute cytotoxic edema in the left substantia nigra (SN) and globus pallidus while T2WI also showed hyperintensity in the same regions. The SN was outside the aforementioned middle cerebral arterial territory, which includes the putamen. These findings are compatible with the diagnosis of acute pallidonigral injury secondary to striatal infarction. The patient had fully recovered from his right-lower-limb weakness after 1 month.

**Conclusions:**

Secondary pallidonigral degeneration may mimic recurrent stroke. DWI along with T2WI facilitates elucidation of this clinicopathological entity, and thus unnecessary treatment can be avoided.

## Background

Secondary pallidonigral degeneration is a rare clinicopathological entity in which excitotoxicity occurs in the neurons of the globus pallidus and substantia nigra (SN) following transneuronal degeneration (TND) caused by prior upstream brain injury, usually in the putamen. Diffusion-weighted imaging (DWI) is sensitive to acute cytotoxic edema in regions with TND because it exhibits reduced apparent diffusion coefficient (ADC) values [1, 2]. Patients with secondary degeneration may present with new or prolonged neurological deficits that could be mistaken for other pathological entities and mislead clinical management [3]. This paper reports a case that emphasizes excitotoxicity and magnetic resonance imaging (MRI) findings.

## Case presentation

A 64-year-old man with a history of hypertension and type 2 diabetes mellitus complained of an acute relapse of right-lower-limb weakness following a cerebral infarction 2 months before presentation. In the emergency room, he presented with reduced muscle power in his right lower extremity and no signs of dysarthria, dysphasia, or facial palsy. Initially, recurrent cerebral ischemia was diagnosed.

**Fig. 1:**
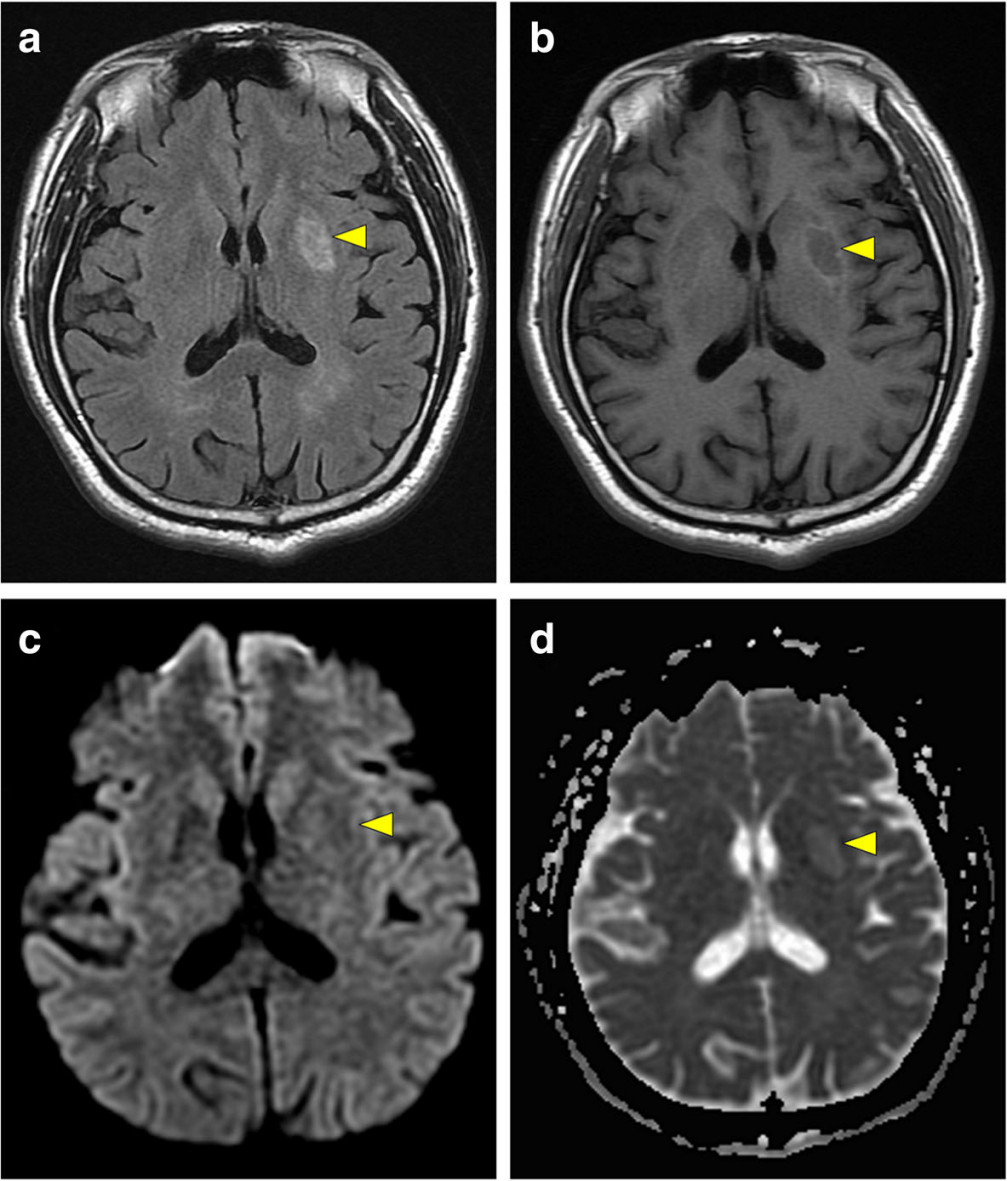
MR scans of the brain at the level of the striatum; FLAIR image (**a**), T1-weighted image (**b**), diffusion-weighted image (**c**), and apparent diffusion coefficient map (**d**), revealing the presence of a subacute to chronic infarct in the left putamen (arrowheads)

**Fig. 2:**
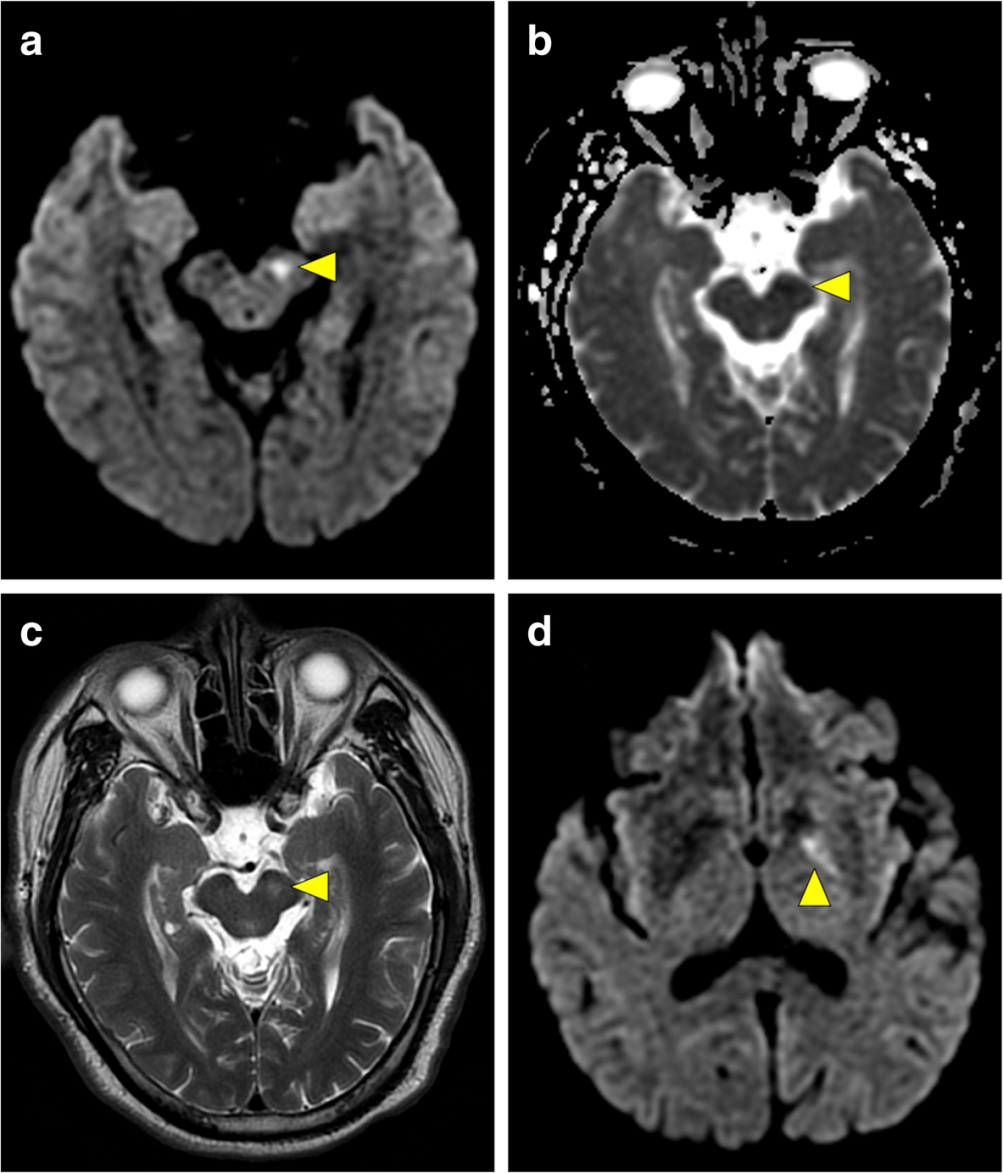
MR scans of the brain at the level of the midbrain and thalamus revealed hyperintense changes on DWI and T2WI in the left SN and globus pallidus (**a**, **c** and **d**, arrowheads) with corresponding low signal intensity on the apparent diffusion coefficient map (**b**, arrowhead). The findings indicated acute pallidonigral degeneration secondary to striatonigral pathway disruption

MR scans of the brain (GE healthcare, Signa HDxt 1.5 T) were obtained on the same day; the scans showed an old infarct in the left putamen with hyperintensity on fluid-attenuated inversion recovery (FLAIR), T2-weighted imaging (T2WI), and the ADC map (Fig. 1), which was consistent with the patient’s recent stroke history. However, MRI revealed an additional high signal intensity on DWI and low signal intensity on the ADC map in the left SN and globus pallidus, which were suggestive of acute cytotoxic edema (Fig. 2). Moreover, hyperintensity was also observed in the same regions on FLAIR/T2WI, findings that are unusual for acute ischemic stroke. Because the left SN was outside the middle cerebral arterial territory, the findings were most compatible with a diagnosis of acute pallidonigral injury secondary to striatal infarction. The patient received conservative treatment and gradually recovered right-lower-limb strength over a 1-month period.

## Discussion and conclusions

Secondary pallidonigral degeneration outside the middle cerebral artery (MCA) territory after an ipsilateral MCA territory infarct has been reported in previous studies using conventional MRI at later stages [4–6]. Such TNDs, which include focal neuronal death, gliosis, and axonal degeneration in chronic cases, mainly occur some weeks after the primary onset of stroke and may be mistaken for a new episode of cerebral infarction. This is particularly true when ischemic-sensitive DWI is used in routine stroke protocol because similar DWI water-restriction findings in the TND may mislead the clinical physician, as reported in our case. Delayed hyperintense lesions in the ipsilateral SN involving the striatal structure could also occur without overt clinical symptomatology even at a later stage or after the development of ischemic lesions [2]. The clinical manifestation in patients with secondary pallidonigral degeneration has not been well elucidated in previous reports. Our case is the first report of secondary pallidonigral degeneration manifested as stroke mimics.

SN is a vulnerable central nervous system structure that is vulnerable to injury caused by secondary degeneration. Previous reports have shown neuronal loss in the compact zone of the SN after massive unilateral basal ganglia infarction [6]. This type of insult has been attributed to excessive excitation of the postsynaptic deep nuclei due to the loss of trans-synaptic γ-aminobutyric acid (GABA-)ergic inputs, which mediate the striatonigral pathway [6, 7]. In our patient, the observed cytotoxic edema in the pallidum and SN, as shown in DWI, were likely due to axonal intramyelinic swelling or astrocytic swelling [8, 9], a result of disinhibition of the striatonigral GABAergic pathway. The local increase in fluid accumulation within the inter-myelinic layers could also be the underlying cause of MR signal changes on FLAIR/T2WI at acute setting.

In an acute setting, in contrast to ischemia-sensitive DWI, computed tomography incompletely depicts TND. MRI provides accurate anatomical and pathological information regarding primary brain injury and associated secondary lesions. A complete MRI protocol for cerebral stroke, including T1-weighted images, T2-weighted images, T2 FLAIR, DWI, and MRI angiography, not only provides sensitive and specific signal intensity changes in the primary infarcted site but also distinguishes old infarcts from secondary neuronal degeneration. In summary, secondary striatonigral degeneration may mimic recurrent stroke clinically and radiologically. Understanding this nonischemic and excitotoxicity-induced injury can prevent administration of unnecessary treatment to patients.
